# Comment on Cho et al. Effects of Adjuvant Respiratory Therapy on Secretion Expectoration and Treatment Adherence in Patients with Head and Neck Cancer Receiving Concurrent Chemo-Radiotherapy. *Medicina* 2025, *61*, 1266

**DOI:** 10.3390/medicina62030505

**Published:** 2026-03-10

**Authors:** Aybala Nur Ucgul, Serap Catli Dinc

**Affiliations:** Department of Radiation Oncology, Faculty of Medicine, Gazi University, Ankara 06010, Turkey; serapcatli@gmail.com

Congratulations to Cho and colleagues for their study, “Effects of Adjuvant Respiratory Therapy on Secretion Expectoration and Treatment Adherence in Patients with Head and Neck Cancer Receiving Concurrent Chemo-Radiotherapy” [1]. This single-center study investigates the impact of adding respiratory therapy with terbutaline and ipratropium to routine breathing exercises and expel techniques in head and neck cancer patients undergoing concurrent chemoradiotherapy. While the findings indicate an improvement in the completion rate of treatment and secretion expectoration, we would like to raise several concerns that could influence the findings.

A statistically significant gender imbalance was identified between the two groups (*p* = 0.04). Notably, the control group did not include any female patients. It has been established that female patients generally exhibit lower lung volumes and differences in airway mechaniscs, which may influence mucociliary clearance, particularly under the additional physiological stress of concurrent chemo-radiotherapy [2]. Therefore, it can be suggested that gender may be a factor influenced by mucus secretion. Moreover, significant respiratory diseases such as chronic obstructive pulmonary disease, a leading cause of mucus hypersecretion, were not documented. This omission represents a significant limitation in the interpretation of the outcomes [3].

Another major limitation is the paucity of detailed information regarding the primary tumor site, the radiotherapy dose, the irradiated volume and the chemotherapy regimen. This deficiency has the potential to influence the outcomes.

It is important to note that while patients in the research group executed their breathing exercises under the direct supervision of a respiratory therapist during each session, patients in the control group performed these exercises on their own, without professional guidance. This difference may have affected treatment adherence. Furthermore, it remains uncertain whether control group patients performed these exercises regularly or correctly, which limits the reliability of the comparisons between groups. Future studies could minimize this limitation by implementing standardized patient education protocols and periodic supervised reinforcement sessions to ensure consistency in exercise performance across study groups.

Moreover, the statistical analyses in this study are difficult to evaluate due to the limited methodological details provided. The authors do not clarify how they compared categorical outcomes, and it is unclear whether they verified the assumptions for applying parametric tests, such as using the Student’s *t*-test for Likert-scale data. This lack of information makes it difficult to judge the reported *p*-values and the robustness of the conclusions.

Beyond these methodological concerns, the study is confined in its scope to subjective outcomes, including patient-reported symptom relief and treatment adherence, omitting the assessment of more objective or clinically significant endpoints, such as pneumonia or repiratory-related hospitalizations. Finally, the utilization of self-reported questionnaires, absent blinding or objective verification, introduces an additional risk of bias in the evaluation of the reported benefits of the intervention.

In conclusion, while the authors emphasize a critical component of supportive care in this patient population, the limitations outlined above necessitate a cautious interpretation of their findings. We believe that future prospective studies with better design, appropriate statistical analyses and objective clinical endpoints are needed to validate the role of adjuvant respiratory therapy in this setting.
